# CXCL12/CXCR4/CXCR7 axis in placenta tissues of patients with placenta previa

**DOI:** 10.1515/biol-2022-0642

**Published:** 2023-08-11

**Authors:** Xia Wu, Ying Wang, Min Li

**Affiliations:** Department of Obstetrics, Maternal and Child Health Hospital of Hubei Province, Wuhan 430070, Hubei, China

**Keywords:** CXCL12, CXCR4, CXCR7, placenta previa, placental tissue, human trophoblast cells

## Abstract

CXCR4 and CXCR7 have been revealed to be receptors of CXCL12. This research was designed to probe the expression of chemokine CXCL12 and its receptors CXCR4 and CXCR7 in placental tissues of patients with placenta previa and the effect of CXCL12/CXCR4/CXCR7 axis on the biological functions of human trophoblast cells. CXCL12, CXCR4, and CXCR7 expression in placental tissue from patients with placenta previa and healthy puerperae was detected. CXCL12, CXCR4, and CXCR7 expression in human trophoblast cell lines (HTR8/SVneo cells) was assessed after suppression or overexpression of CXCL12, CXCR4, and CXCR7. The cell proliferative, invasive, and migratory capacities were also evaluated in HTR8/SVneo cells after suppression or overexpression of CXCL12, CXCR4, and CXCR7. CXCL12, CXCR4, and CXCR7 expression was elevated in placental tissues from patients with placenta previa. Downregulation of CXCL12, CXCR4, and CXCR7 could lead to decreased mRNA levels of CXCL12, CXCR4, and CXCR7 in HTR-8/SVneo cells, which was accompanied by diminished cell proliferative, migratory, and invasive capabilities. Overexpression of CXCL12, CXCR4, and CXCR7 genes presented an opposite tendency. CXCL12, CXCR4, and CXCR7 are highly expressed in placental tissues of patients with placenta previa and induce the biological activities of HTR8/SVneo cells.

## 1 Introduction

Placenta previa is a condition where the placenta is implanted in the vascularized lower uterine segments, and it may lead to inadequate uteroplacental perfusion, which adversely impacts the neonatal outcome [1]. Placenta previa results from multiple risk factors, such as multiparity, multiple gestations, advanced maternal age, maternal cigarette smoking, and especially, cesarean section history [2]. Generally, women with placenta previa have a high risk of requiring postpartum emergent hysterectomy, blood transfusions, as well as the elevated duration of hospitalization postpartum [3,4].

In recent years, achievements in grayscale and Doppler ultrasound have made a favor for the prenatal diagnosis of abnormal placentation [5]. The combination of proactive management with a multidisciplinary approach is able to diminish hemorrhage and allow for appropriate surgery, indicating a low maternal and fetal incidence, and maintained fertility [6,7]. Hence, it is urgent for the exploration of more biomarkers for the improvement of the placenta previa diagnosis and therapy.

Chemokines, together with their receptors, are essential in the maternal immune response with their expression levels at the maternal–fetal interface, which participate in leukocyte migration, angiogenesis, as well as cell activation [8]. Chemokine ligand 12 (CXCL12) is an 8 kDa chemokine belonging to the CXC subgroup. CXCR4 and CXCR7 have been revealed to be receptors of CXCL12 [9]. It is reported that CXCL12 and its receptors CXCR4/CXCR7 participate in diverse physiological processes (e.g., inflammatory response, tumorigenesis, as well as cell activities) via the activation of the downstream signal pathways [10]. For instance, Zhu et al. have found that CXCL12 intensifies the survival of human neural progenitor cells via the CXCR4- or CXCR7-mediated endocytotic pathway [11]. In the meanwhile, evidence has shown the relationships of CXCL12, CXCR4, and CXCR7 with trophoblastic cell apoptosis, and these factors may be linked to preeclampsia (PE) [12]. Furthermore, Leavey et al. have made an extensive transcriptome analysis of PE versus control placenta. Their dataset suggests significant variations for CXCR4 (increased in PE) and CXCR6 (decreased in PE), and CXCL4 and CXCL6, both induced in PE at the transcriptome level, which could substantiate the idea that the CXCR/CXCL system might be involved in placental disease [13]. As shown above, the CXCL12/CXCR4/CXCR7 axis is of great significance in various physiological and pathological conditions, but their functions in placenta previa remain largely unknown. HTR-8/SVneo cells have a similar phenotype compared to their primary cell counterparts *in vivo* [14] and are readily available, so HTR-8/SVneo cells were used as the target cells in this study. Consequently, this work intended to probe the expression of chemokine CXCL12 and its receptors CXCR4 and CXCR7 in placental tissues of patients with placenta previa and the effect of CXCL12/CXCR4/CXCR7 axis on the biological functions of human trophoblast cells, providing an experimental basis for placenta previa therapy.

## 2 Materials and methods

### 2.1 Participants

From July 2019 to January 2021, 42 puerperae with placenta previa who had cesarean section (placenta previa group) and 42 normal puerperae who had cesarean section (Control group) were admitted into the Obstetrics Department of Maternal and Child Health Hospital of Hubei Province. Among the 42 patients with placenta previa, there were 20 patients with total placenta previa, 12 patients with partial placenta previa, and 10 patients with marginal placenta previa. The basic information is listed in Table 1.

**Table 1:** Basic information of the puerperae

General information	Placenta previa group (*n* = 42)	Control group (*n* = 42)	*P* value
Age (y)	29.43 ± 3.95	29.23 ± 3.91	0.816
BMI (kg/m^2^)	27.05 ± 3.23	26.49 ± 3.40	0.444
Gestational week (w)	36.23 ± 1.31	36.34 ± 1.51	0.700
Neonatal weight (kg)	2.71 ± 0.42	2.85 ± 0.35	0.090
Smoking history			0.757
Yes	5	7	
No	37	35	
Birth history			0.110
Yes	19	11	
No	23	31	
Scar uterus			0.026
Yes	10	2	
No	32	40	
History of pelvic inflammation			0.007
Yes	12	2	
No	30	40	
History of cesarean section			0.049
Yes	16	7	
No	26	35	
History of abortion			0.028
Yes	25	14	
No	17	28	
Subtypes of placenta previa			
Total placenta previa	20		
Partial placenta previa	12		
Marginal placenta previa	10		

Note: y, year; w, week; BMI, body mass index.

The patients with placenta previa aged 21–42 years old, cooperated with clinical medical work; had a singleton pregnancy and 1–10 pregnancies, as well as chose caesarean section, were enrolled in our experiment. The patients were diagnosed with placenta previa by type B ultrasound, vaginal examination, cesarean section, or transvaginal delivery after 28 weeks of pregnancy. The patients were excluded if they had a clear rupture and severe bleeding in the fetal membrane and required immediate intervention, presented with unstable fetal heart rate, intrauterine fetal death or major fetal malformation, abortion, placental abruption, and known hemorrhagic diseases, and had potential chronic hypertension, diabetes, kidney disease, blood disease, heart disease or any medical disease [15].


**Informed consent:** Informed consent has been obtained from all individuals included in this study.
**Ethical approval:** The research related to human use has been complied with all the relevant national regulations, institutional policies and in accordance with the tenets of the Helsinki Declaration, and has been approved by the Ethics Committee of Maternal and Child Health Hospital of Hubei Province (approval number: 20190415).

### 2.2 Placental collection

Immediately after delivery of the placenta from a woman who had undergone cesarean section, several pieces of placenta tissues of 1.0 cm × 1.0 cm × 1.0 cm in size were taken from the central part of the placenta on the maternal side (avoiding necrosis, calcification points, and bleeding areas when cutting), and rinsed repeatedly in saline. About 100 mg of them were packed into RNA enzyme inactivated-Eppendorf (EP) tubes and stored in a refrigerator at −70°C for subsequent RT-qPCR assay. The placental tissues were fixed in 10% formalin solution for 24–48 h, embedded in conventional paraffin, and serially sectioned at 4 μm thickness for immunohistochemical detection.

### 2.3 Cell culture

The human trophoblast cell line (HTR-8/SVneo) was acquired from the American Type Culture Collection (Manassas, VA, USA; CRL-3271), which was cultivated in RPMI-1640 (Gibco, Grand Island, NY, USA) encompassing 5% fetal bovine serum (FBS, Gibco), along with penicillin/streptomycin (1,000 U/mL, Gibco) [16].

### 2.4 RT-qPCR analysis

RNA from placental samples or HTR-8/SVneo cells was extracted with the TRIzol reagent (Invitrogen, Carlsbad, CA, USA), which was reverse-transcribed into a cDNA with a High-Capacity cDNA Reverse Transcription kit (Thermo Fisher Scientific, Waltham, MA, USA). The qPCR was implemented with the ABI PRISM 7700 system, together with FastStart Universal SYBR Green Master kit (Roche Diagnostics, Mannheim, Germany). The detailed primer sequences included in this study are shown in Table A1. Relative quantification of mRNA was conducted using the comparative 2^−ΔΔCt^ method [17] with GAPDH as the reference gene. The formula was as follows: ΔΔCt = [Ct(target gene) − Ct(reference gene)]_experimental group_ − [Ct(target gene) − Ct(reference gene)]_control group_.

### 2.5 Immunohistochemistry (IHC)

Paraffin-embedded sections of placental tissues were prepared, heated at 65℃ for 2 h, and dewaxed in xylene I and II. Afterward, sections were treated with 100, 95, 85, and 70% ethanol, immersed for 2 min in boiled 0.01 M citrate buffer, cooled for 30 min and subsequently rinsed with phosphate buffered saline. Subsequently, sections were cultivated for 20 min in 3% H_2_O_2_ in order to block the endogenous peroxidase activity. Next, sections were blocked by normal goat serum and cultivated at 4℃ overnight with primary antibodies (CXCL12, 1:100; CXCR4, 1:100; CXCR7, 1:200; all from R&D). Lastly, sections were subjected to incubation with MaxVision^TM^ HRP-Polymer anti-mouse IHC Kit (Maixin, Fuzhou, China), DAB development, alcohol gradient dehydration, xylene immersing, neutral gum blocking, as well as observation under a microscope. A double-blind method was utilized to judge each section independently. The images were collected and saved, the immunohistochemical results were analyzed using Image-Pro-Plus image analysis software (Media Cybernetics, Bethesda, MD, USA), and the integrated optical density (IOD) values were calculated. IHC results were interpreted according to the following criteria: when the cell membrane or cytoplasm showed brownish-yellow particles, the cells were positive.

### 2.6 Cell grouping and transfection

HTR-8/SVneo cells were grouped as follows: Blank group (without transfection), overexpression-negative control (OE-NC) group, short hairpin RNA-NC (sh-NC) group, OE-CXCL12 group, OE-CXCR4 group, OE-CXCR7 group, sh-CXCL12 group, sh-CXCR4 group, and sh-CXCR7 group. The aforesaid overexpression and interference lentiviruses were obtained from RiBoBio (Guangzhou, China). HTR-8/SVneo cells were cultivated at 2 × 10^5^ per well in six-well plates. The thawed virus liquid was blended with a complete medium with 10 μg of polycoagulide. The medium in the plates was discarded and the remaining mixture was cultivated with a diluted viral mixture for 24 h, followed by a supplement of the complete medium for growth overnight. The puromycin was added 96 h after lentiviral infection of cells, and after screening, cells were passaged to 25 cm^2^ culture flasks. After 2 weeks of puromycin discontinuation, a portion of the cell samples were collected for RNA and protein extraction, and another portion were used for subsequent experiments.

### 2.7 Cell proliferation assay

The conduction of the cell proliferation assay was realized using Cell Counting Kit-8 (CCK-8) (Beyotime, Shanghai, China). The absorbance of each well was measured at the OD450 wavelength with the application of an automated microplate reader [18].

### 2.8 Cell invasion assay

We used a Transwell membrane (8 µm pore size; BD Biosciences, Bedford, MA, USA) coated with Matrigel (BD Biosciences) for invasion assay. Cells were grown to 60–80% confluence after trypsinization. Cells (2 × 10^5^, 100 μL) in the serum-free RPMI-1640 were put into the upper chamber of each well of a 24-well transwell polycarbonate membrane (8 µm pore size) coated with 50 µL Matrigel. The RPMI-1640 medium (600 μL) containing 10% FBS served as a chemoattractant, was put into the lower chambers. After wells were incubated for 24 h at 37°C, the non-invaded cells in the upper compartment were removed and the chamber was washed twice with PBS. The cells that had invaded through the membrane were stained with methanol and 0.1% crystal violet, imaged, and counted using an inverted microscope (Olympus, Tokyo, Japan) and quantified from visualizing five random fields at a magnification of ×400

### 2.9 Cell migration assay

Uniform horizontal lines were drawn with a marker on a six-well plate, with at least five horizontal lines passing through each well, with both lines separated by 0.5–1 cm. About 5 × 10^5^ cells were supplemented to each well in the well plate, and 24 h later, 200 μL sterile pipette tip was used to make a scratch on the cells perpendicular to the horizontal line on the back. Cells were appended with an appropriate serum-free medium and placed in an incubator for the visualization of the wound healing process. The average distance between cells after 48 h was calculated with Image J software.

### 2.10 Statistical analysis

SPSS 21.0 software was adopted for statistics. Measurement data, depicted as mean ± standard deviation, were processed by the *t*-test or one-way analysis of variance. Enumeration data, expressed by percentage or rate, were processed by Fisher’s exact test. A significant difference was witnessed if *P* value <0.05.

## 3 Results

### 3.1 Basic information of the puerperae

The basic information of puerperae is exhibited in Table 1. No difference was observed in age, body mass index, gestational week, smoking history, birth history, and neonatal weight between the placenta previa and the control groups (all *P* > 0.05), and a significant difference was witnessed in the cases of scar uterus, history of pelvic inflammation, history of cesarean section, and history of abortion (all *P* < 0.05).

### 3.2 Relationship between levels of chemokines CXCL12, CXCR4, and CXCR7 and clinical parameters of placenta previa

The relationship between the expression levels of CXCL12, CXCR4, and CXCR7 mRNA in placental tissues of the patients with placenta previa and their clinical parameters was analyzed. The findings demonstrated that the levels of CXCL12, CXCR4, and CXCR7 in placental tissues of the patients with placenta previa exhibited no difference in age, smoking history, and birth history (all *P* > 0.05), and a significant difference was witnessed in the cases of scar uterus, history of pelvic inflammation, history of cesarean section, and history of abortion (all *P* < 0.05) (Table 2).

**Table 2:** Relationship between levels of chemokines CXCR4, CXCR7, and CXCL12 and clinical parameters of placenta previa

General information	Case	CXCL12	*P* value	CXCR4	*P* value	CXCR7	*P* value
Age (y)			0.087		0.051		0.179
≥30	22	1.65 ± 0.20		2.36 ± 0.22		2.97 ± 0.29	
<30	20	1.46 ± 0.45		2.10 ± 0.55		2.74 ± 0.76	
Smoking history			0.503		0.666		0.479
Yes	5	1.46 ± 0.02		2.16 ± 0.03		2.69 ± 0.08	
No	37	1.57 ± 0.37		2.25 ± 0.46		2.88 ± 0.60	
Birth history			0.082		0.051		0.087
Yes	19	1.66 ± 0.22		2.38 ± 0.22		3.03 ± 0.29	
No	23	1.48 ± 0.41		2.12 ± 0.52		2.72 ± 0.70	
Scar uterus			0.008		0.008		0.036
Yes	10	1.81 ± 0.12		2.55 ± 0.11		3.19 ± 0.14	
No	32	1.48 ± 0.36		2.14 ± 0.45		2.76 ± 0.61	
History of pelvic inflammation			0.020		0.037		0.016
Yes	12	1.76 ± 0.50		2.46 ± 0.62		3.19 ± 0.84	
No	30	1.48 ± 0.24		2.15 ± 0.30		2.73 ± 0.36	
History of cesarean section			0.044		0.032		0.029
Yes	16	1.70 ± 0.46		2.42 ± 0.56		3.10 ± 0.75	
No	26	1.48 ± 0.23		2.13 ± 0.29		2.71 ± 0.37	
History of abortion			0.047		0.021		0.011
Yes	25	1.65 ± 0.40		2.36 ± 0.47		3.04 ± 0.63	
No	17	1.43 ± 0.22		2.06 ± 0.29		2.60 ± 0.34	

Note: y, year.

### 3.3 CXCL12, CXCR4, and CXCR7 are expressed at a high level in placental tissues of the patients with placenta previa

**Figure 1:**
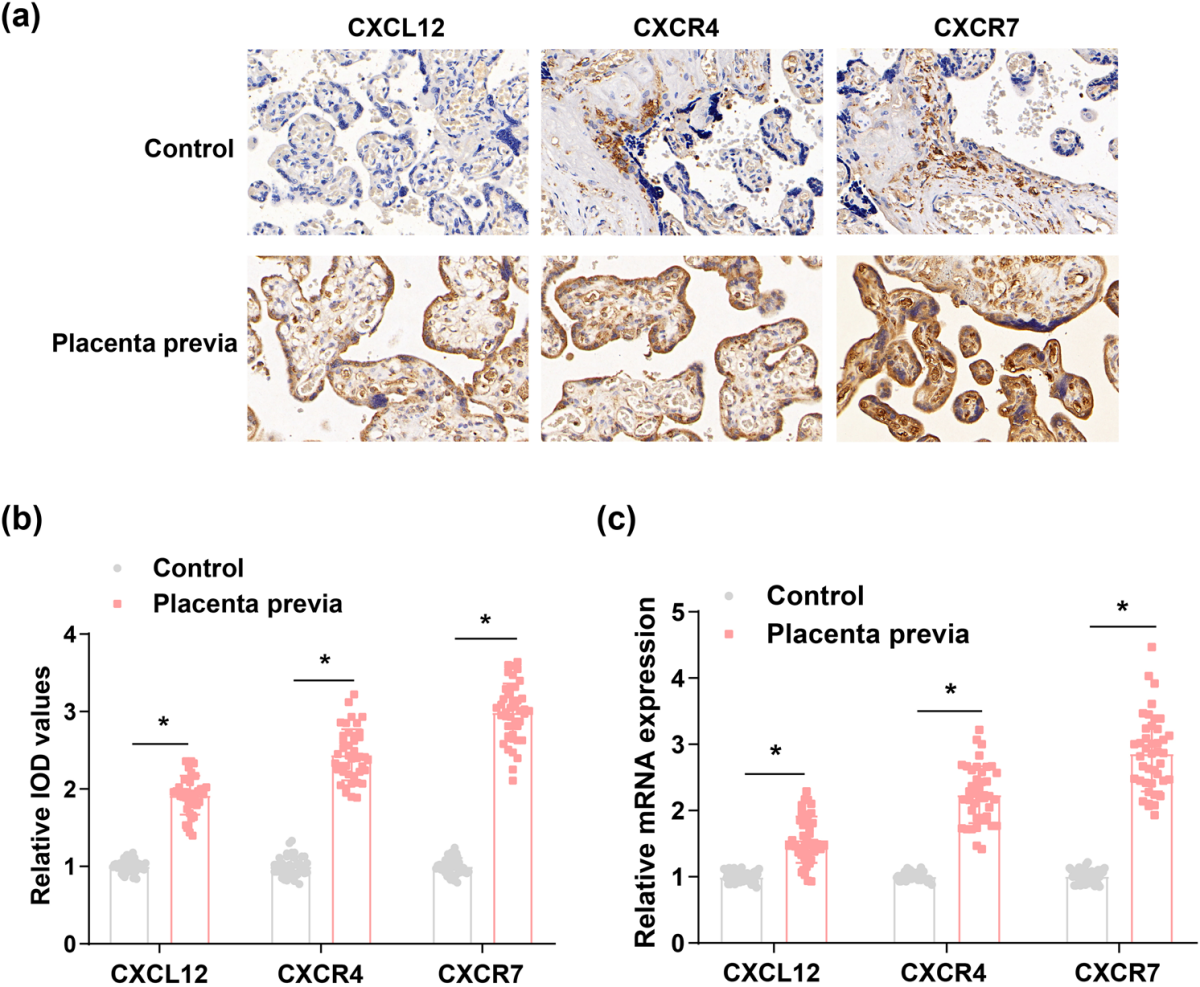
CXCL12, CXCR4, and CXCR7 are expressed at a high level in placental tissues of the puerperae with placenta previa. (a) and (b) CXCL12, CXCR4, and CXCR7 levels in placental tissues were measured by IHC. (c) CXCL12, CXCR4, and CXCR7 mRNA levels in placental tissues were also evaluated by RT-qPCR assay. *P < 0.05.

As reported, the CXCL12/CXCR4/CXCR7 axis can strengthen the cross-talk between trophoblast cells and decidual cells, participate in the differentiation and invasion of trophoblast cells, as well as placental angiogenesis [19]. CXCL12, CXCR4, and CXCR7 levels in placental tissues were evaluated by IHC (Figure 1a and b), and the findings demonstrated that brownish-yellow staining was seen in the cytoplasm of both syncytiotrophoblasts and cytotrophoblasts in the placental tissues of pregnant women with placenta previa. Meanwhile, the IOD values of CXCL12, CXCR4, and CXCR7 IHC results were analyzed using Image-Pro-Plus software, and the relative IOD values were calculated. The IHC results indicated that the expression of CXCL12, CXCR4, and CXCR7 in placenta tissues of patients with placenta previa was higher than those of normal puerperae. In the meantime, CXCL12, CXCR4, and CXCR7 mRNA levels in placental tissues were also evaluated by RT-qPCR assay, which elucidated that high CXCL12 and its receptor CXCR4 and CXCR7 levels were witnessed in the placental tissues of patients with placenta previa in comparison to those in the placental tissues of normal puerperae (Figure 1c).

### 3.4 Establishment of a human trophoblast HTR-8/SVneo cell line that either inhibit or overexpresses the CXCL12, CXCR4, and CXCR7 genes

**Figure 2:**
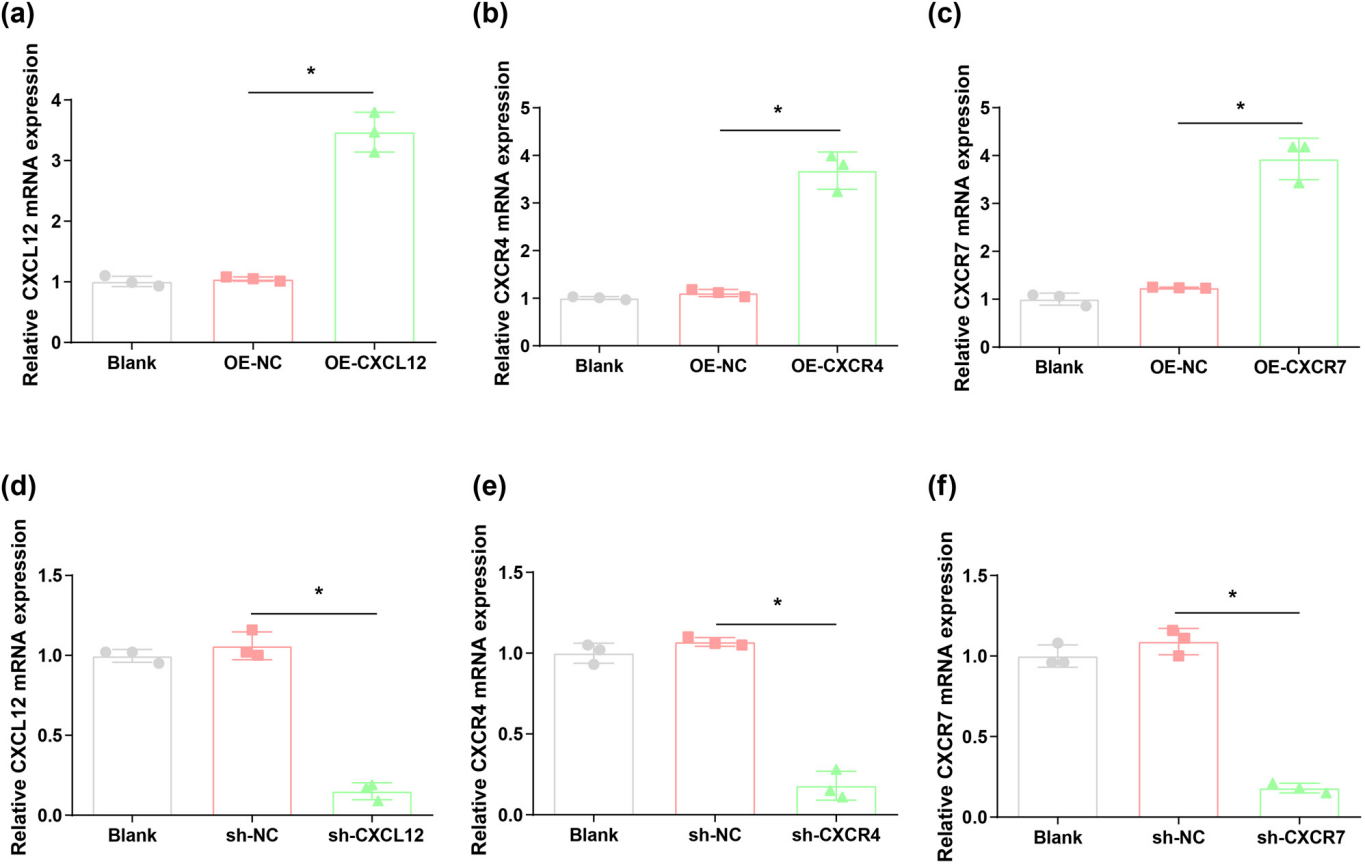
CXCL12, CXCR4, and CXCR7 expression in human trophoblast HTR-8/SVneo cells silencing or overexpressing CXCL12, CXCR4, and CXCR7. (a–c) mRNA expression levels of CXCL12, CXCR4, and CXCR7 were measured in cells overexpressing CXCL12, CXCR4, and CXCR7 by qRT-PCR. (d–f) mRNA expression levels of CXCL12, CXCR4, and CXCR7 were evaluated in cells silencing CXCL12, CXCR4, and CXCR7 by RT-qPCR. *P < 0.05.

To detect the efficiency of transfection, RT-qPCR was implemented for verifying the expression of CXCL12, CXCR4, and CXCR7 mRNA in human trophoblast HTR-8/SVneo cells after interference or overexpression. Results from RT-qPCR assay demonstrated that the expressions of CXCL12, CXCR4, and CXCR7 in HTR-8/SVneo cells were increased after overexpression with OE-CXCL12, OE-CXCR4, and OE-CXCR7, respectively (Figure 2a–c). On the contrary, the levels of CXCL12, CXCR4, and CXCR7 were decreased in HTR-8/SVneo cells treated with sh-CXCL12, sh-CXCR4, and sh-CXCR7, respectively (Figure 2d–f).

### 3.5 Overexpression of CXCL12, CXCR4, and CXCR7 genes induce the HTR-8/SVneo cell proliferation

**Figure 3:**
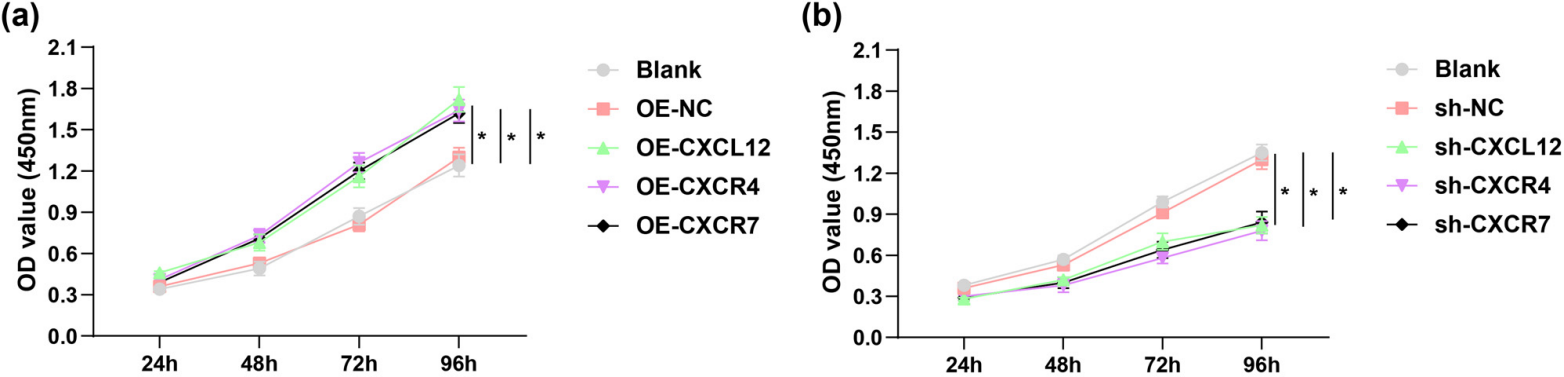
Effects of suppression or overexpression of CXCL12, CXCR4, and CXCR7 on HTR-8/SVneo cell viability. (a) CCK-8 assay for evaluating the roles of overexpressing CXCL12, CXCR4, and CXCR7 in HTR-8/SVneo cell viability (b) CCK-8 assay for evaluating the roles of silencing CXCL12, CXCR4, and CXCR7 in HTR-8/SVneo cell viability. *P < 0.05.

CCK-8 assay for evaluating the roles of CXCL12, CXCR4, and CXCR7 in HTR-8/SVneo cell proliferation elucidated that HTR-8/SVneo cell proliferation was enhanced after the expressions of CXCL12, CXCR4, or CXCR7 genes were overexpressed (Figure 3a). On the contrary, the cell proliferative capacity of HTR-8/SVneo cells was decreased after the expression levels of CXCL12, CXCR4, or CXCR7 genes were silenced (Figure 3b).

### 3.6 Overexpression of CXCL12, CXCR4, and CXCR7 genes promote the HTR-8/SVneo cell migratory and invasive capacities

**Figure 4:**
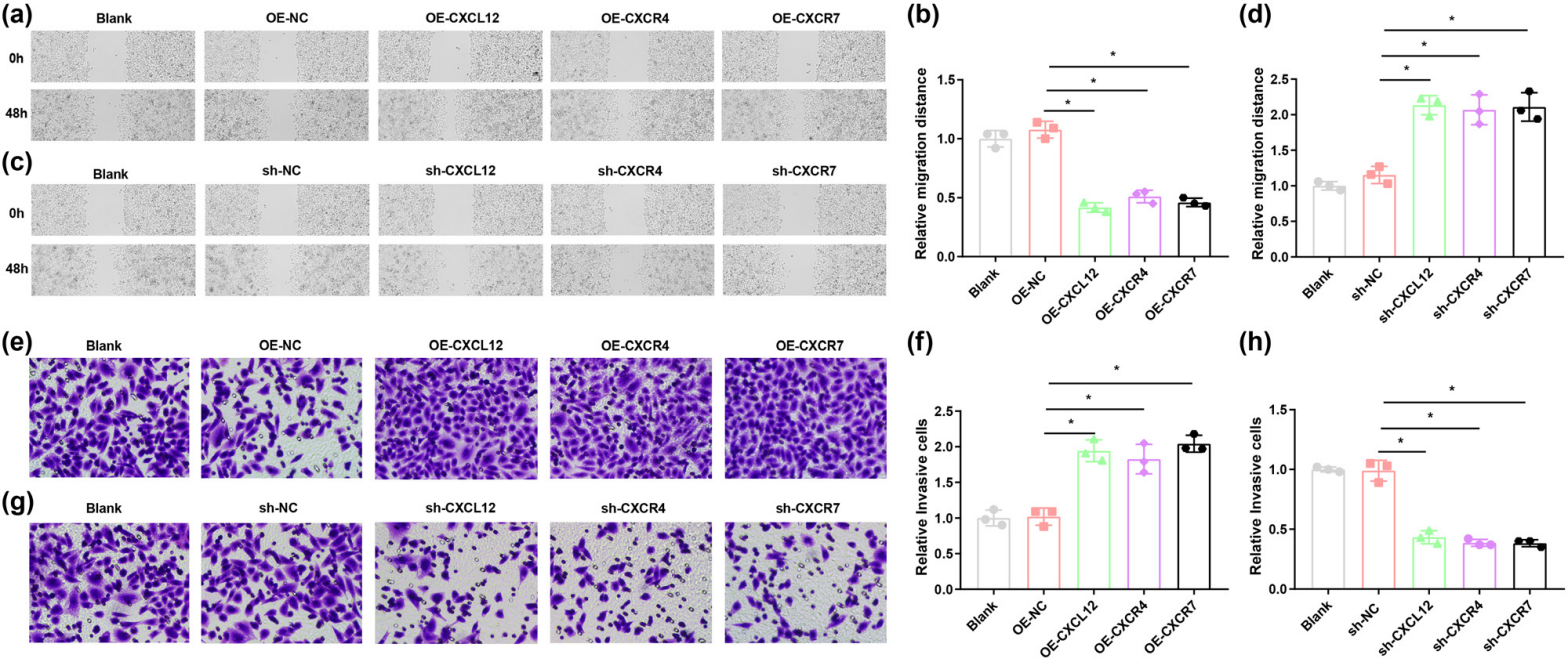
Effect of silencing or restoration of CXCL12, CXCR4, and CXCR7 on the migratory and invasive capacities of human trophoblast HTR-8/SVneo cells. (a) and (b) Cell scratch test was implemented for assessing the HTR-8/SVneo cell migration capability after overexpression of CXCL12, CXCR4, and CXCR7. (c) and (d) Cell scratch test was implemented for assessing the HTR-8/SVneo cell migration capability after suppression of CXCL12, CXCR4, and CXCR7. (e) and (f) The number of cells crossing the human-like basement membrane in the HTR-8/SVneo cells after overexpression of CXCL12, CXCR4, and CXCR7 was observed microscopically using the Transwell invasion assay. (g) and (h) The number of cells crossing the human-like basement membrane in the HTR-8/SVneo cells after suppression of CXCL12, CXCR4, and CXCR7 was observed microscopically using the Transwell invasion assay. *P < 0.05.

The cell scratch test was implemented for assessing the HTR-8/SVneo cell migration capability after silencing or overexpression of CXCL12, CXCR4, or CXCR7. It was observed that there exhibited reduced migration distance and enhanced cell migratory capacity in the HTR-8/SVneo cells after overexpression of CXCL12, CXCR4, or CXCR7 (Figure 4a and b). Downregulation of CXCL12, CXCR4, or CXCR7 was found to contribute to widened migration distance, and impeded cell migratory capacity in the HTR-8/SVneo cells (Figure 4c and d).

The number of cells crossing the Matrigel-coated basement membrane in the HTR-8/SVneo cells after suppression and overexpression of CXCL12, CXCR4, or CXCR7 was observed microscopically using the Transwell invasion assay. It was observed that there exhibited an elevated number of cells crossing the Matrigel-coated basement membrane and enhanced cell invasive capacity in the HTR-8/SVneo cells after overexpression of CXCL12, CXCR4, and CXCR7 (Figure 4e and f). Downregulation of CXCL12, CXCR4, or CXCR7 was found to contribute to a reduced number of cells crossing the Matrigel-coated basement membrane and impeded cell invasive capacity in the HTR-8/SVneo cells (Figure 4g and h).

## 4 Discussion

Placenta previa is a leading reason of maternal morbidity and mortality, and it is linked to a high risk of perinatal bleeding and hysterectomy [20]. The complexity of placenta previa is dependent on newly formed vessels, tissue destruction, as well as vascular invasion of surrounding tissues, and requires multi-disciplinary management [21]. At present, the exact biochemical markers of placenta previa have been discussed, and the early diagnosis of placenta previa in the puerperae can reduce the risk of the complications. This research was designed to probe the levels of the chemokine CXCL12 and the receptors CXCR4/CXCR7 in the placental tissues of patients with placenta previa and their effects on the biological functions of human trophoblast cells. These findings demonstrated that overexpression of CXCL12, CXCR4, and CXCR7 induced the biological activities of HTR8/SVneo cells.

Chemokines can bind to multiple receptors that collectively constitute a complicated network, and these chemokines are implicated in cellular immunity, inflammation, growth, as well as other physiological functions [22]. Particularly, CXCL12 binds to its cognate receptors (CXCR4 and CXCR7) with a high affinity, which mediates disease progression through inducing the activation of the downstream pathways [23]. In our study, we observed that there was a CXCL12 level in the placental tissues of puerperae with placenta previa, and elevated CXCL12 induced the HTR8/SVneo cell activities. CXCL12 is activated in the process of implantation and placentation, revealing a vital role in the communication between the maternal endometrium and trophoblast cells [24]. Meanwhile, the differential localization of CXCL12 in the glandular endometrium suggests that CXCL12, secreted by uterine glands, could support placental development and fetal survival in a paracrine and/or autocrine manner [25]. The aforesaid findings confirm the participation of CXCL12 in gynecological diseases.

Increasing evidence has focused on the combined actions of the chemokine CXCL12 and the receptors CXCR4/CXCR7 in different female diseases. For instance, the CXCL12/CXCR4 is a particular pair of chemokine/chemokine receptors that plays a part in placentation, implantation, along with embryogenesis [24]. It is reported that the CXCL12/CXCR4 biological axis induces human trophoblast cell viability by activating EGFR and the ERK pathway [26,27]. Besides, the CXCL12/CXCR4 axis can control the decidual mesenchymal stem cell migratory behaviors, which function in the PE process [28]. Also, Liao et al. have supported that CXCL12 can be combined with CXCR7 to activate downstream signaling molecules, to promote cell survival and proliferation [29]. Balabanian et al. have stated that CXCL12 participates in lymphocyte motility through binding and signaling through CXCR7 which is expressed on the primary T cell surface [30]. Similarly, in our work, we found high CXCR4 and CXCR7 levels in the placental tissues of puerperae with placenta previa, and silencing of CXCR4 and CXCR7 led to diminished HTR8/SVneo cell proliferative, migratory, and invasive capabilities. As previously described, CXCL12, CXCR4, and CXCR7 are expressed in placental tissue in the process of all pregnancy trimesters, and these factors are essential in early pregnancy by an autocrine manner [31].

In summary, we highlight that upregulated CXCL12, CXCR4, and CXCR7 induce the biological activities of HTR8/SVneo cells. Additionally, the levels of CXCR4, CXCR7, and CXCL12 in placental tissues of patients with placenta previa exhibited a significant difference in the cases of scar uterus, history of pelvic inflammation, history of cesarean section, and history of abortion. These findings imply that CXCR4, CXCR7, and CXCL12 are essential parameters in the placenta previa development, and may be potent indicators for the diagnosis of placenta previa. However, a better recognition of the capabilities of the CXCL12/CXCR4/CXCR7 axis in the regulation of trophoblast functions will help to identify potent treatments for female diseases.

## Appendix

**Table A1:** Primers used in the RT-qPCR analysis

Gene abbreviation	Primer sequence	*T*_m_ (°C)	Amplicon size (bp)	Efficiency (%)
CXCL12	Forward: 5′-ATTCTCAACACTCCAAACTGTGC-3′	64	88	98
	Reverse: 5′-ACTTTAGCTTCGGGTCAATGC-3′			
CXCR4	Forward: 5′-GGTGGTATGTTGGCGTCTG-3′	64	194	94
	Reverse: 5′-ATAGCAGGACAGGATGACAATACC-3′			
CXCR7	Forward: 5′-CTATGACACGCACTGCTACATC-3	64	91	96
	Reverse: 5’-CTGCACGAGACTGACCACC-3’			
GAPDH	Forward: 5′-TGACTTCAACAGCGACACCCACT-3′	67	240	93
	Reverse: 5′-GACTGAGTGTGGCAGGGACT-3′			
